# HCV induces transforming growth factor β1 through activation of endoplasmic reticulum stress and the unfolded protein response

**DOI:** 10.1038/srep22487

**Published:** 2016-03-01

**Authors:** Pattranuch Chusri, Kattareeya Kumthip, Jian Hong, Chuanlong Zhu, Xiaoqiong Duan, Nikolaus Jilg, Dahlene N. Fusco, Cynthia Brisac, Esperance A. Schaefer, Dachuan Cai, Lee F. Peng, Niwat Maneekarn, Wenyu Lin, Raymond T. Chung

**Affiliations:** 1 Liver Center and Gastrointestinal Division, Department of Medicine, Massachusetts General Hospital, Harvard Medical School, Boston, MA 02114, USA; 2 Department of Microbiology, Faculty of Medicine, Chiang Mai University, Chiang Mai 50200, Thailand; 3 Department of Infectious Diseases, The First Affiliated Hospital of Nanjing Medical University, Nanjing, Jiangsu, China; 4 Institute of Blood Transfusion, Chinese Academy of Medical Sciences and Peking Union Medical College, Chengdu, China; 5 Department for infectious diseases, The second affiliated hospital of Chongqing medical university, Chongqing 400010, China

## Abstract

HCV replication disrupts normal endoplasmic reticulum (ER) function and activates a signaling network called the unfolded protein response (UPR). UPR is directed by three ER transmembrane proteins including ATF6, IRE1, and PERK. HCV increases TGF-β1 and oxidative stress, which play important roles in liver fibrogenesis. HCV has been shown to induce TGF-β1 through the generation of reactive oxygen species (ROS) and p38 MAPK, JNK, ERK1/2, and NFκB-dependent pathways. However, the relationship between HCV-induced ER stress and UPR activation with TGF-β1 production has not been fully characterized. In this study, we found that ROS and JNK inhibitors block HCV up-regulation of ER stress and UPR activation. ROS, JNK and IRE1 inhibitors blocked HCV-activated NFκB and TGF-β1 expression. ROS, ER stress, NFκB, and TGF-β1 signaling were blocked by JNK specific siRNA. Knockdown IRE1 inhibited JFH1-activated NFκB and TGF-β1 activity. Knockdown of JNK and IRE1 blunted JFH1 HCV up-regulation of NFκB and TGF-β1 activation. We conclude that HCV activates NFκB and TGF-β1 through ROS production and induction of JNK and the IRE1 pathway. HCV infection induces ER stress and the UPR in a JNK-dependent manner. ER stress and UPR activation partially contribute to HCV-induced NF-κB activation and enhancement of TGF-β1.

Over 170 million people worldwide are infected with hepatitis C virus (HCV), which is associated with chronic hepatitis, cirrhosis, and hepatocellular carcinoma^1^,^2^. HCV belongs to genus *Hepacivirus* in the family *Flaviviridae*. As with other positive-stranded RNA viruses, HCV has been shown to induce the formation of a membrane-associated replication complex^3^. HCV proteins have been shown to associate with endoplasmic reticulum (ER) membranes. The envelope glycoproteins (E1, E2) have been shown to associate with ER membranes through their transmembrane domains^4^. In addition, HCV NS4B has been demonstrated to induce the formation of a membranous web^5^,^6^, which derives from the ER membrane^7^ and mediates HCV RNA replication^8^. HCV replication in the ER also likely stimulates the ER stress response pathway^9^. Upon sensing ER stress, HCV-infected cells activate a cytoprotective signaling cascade called the unfolded protein response (UPR)^10^. The UPR is directed by three ER-localized transmembrane proteins, activating transcription factor 6 (ATF6), inositol-requiring protein (IRE1), and protein kinase RNA-like endoplasmic reticulum kinase (PERK). When misfolded proteins accumulate in the ER, ATF6 is translocated from the ER to the Golgi, and then the full-length ATF6 is processed by sphingosine 1-phosphate (S1P) and sphingosine 2-phosphate (S2P) proteases to produce a cytosolic ATF6^11^. This cytoplasmic form of ATF6 is an active transcription factor, which is translocated into the nucleus and induces the transcription of ER stress response genes^12^. Activation of IRE1 by ER stress leads to homodimerization and transphosphorylation of IRE1^13^. The activated IRE1 splices the unspliced X-box protein-1 (XBP-1u) mRNA into its spliced form (XBP-1s). The XBP-1s protein functions as a transcriptional activator of UPR genes that contain the unfolded protein responsive elements (UPREs) and ER-associated degradation (ERAD)-related genes^14^. PERK is an ER-resident transmembrane protein and is activated in a similar manner as IRE1^15^. The activated PERK subsequently phosphorylates eukaryotic translation initiation factor 2α (eIF2α) and leads to inhibition of translation initiation^16^.

Transforming growth factor β (TGF-β1) plays a major pathophysiological role in fibrogenesis and growth dysregulation in cancerous and apoptotic cells^17^. Patients with chronic HCV infection have higher levels of TGF-β1, both total and biologically active forms, compared with healthy controls. Immunohistochemical staining of liver sections revealed that 62% of HCV patients have TGF-β1 present in sinusoidal cells of liver tissue^18^. Elevated serum levels of TGF-β1 have been found in chronic HCV-infected patients, as compared to healthy controls^19^. In addition, HCV core induced TGF-β1 transcription upregulation has been shown in cell lines^20^. HCV JFH-1 infection in Huh-7 cells induces transcriptional stimulation, synthesis, and secretion of bioactive TGF-β1 in a time-dependent manner^21^. We have previously demonstrated that the JFH-1 cell culture infectious HCV induces TGF-β1 through the generation of reactive oxygen species (ROS) and p38 MAPK, JNK, ERK1/2, NFκB-dependent pathways^2^,^22^,^23^. However, the mechanism by which HCV-induced ER stress, UPR activation, and TGF-β1 production are linked has not been fully characterized. We therefore sought to determine whether HCV induces TGF-β1 production through ER stress and UPR activation pathways using the infectious JFH1 HCV replication model. We hypothesized that HCV induction of TGF-β1 is mediated by ER stress and the UPR.

## Results

### HCV infection induces the antioxidant response, ER stress, NFκB, and TGF-β1 gene expression

**Figure 1:**
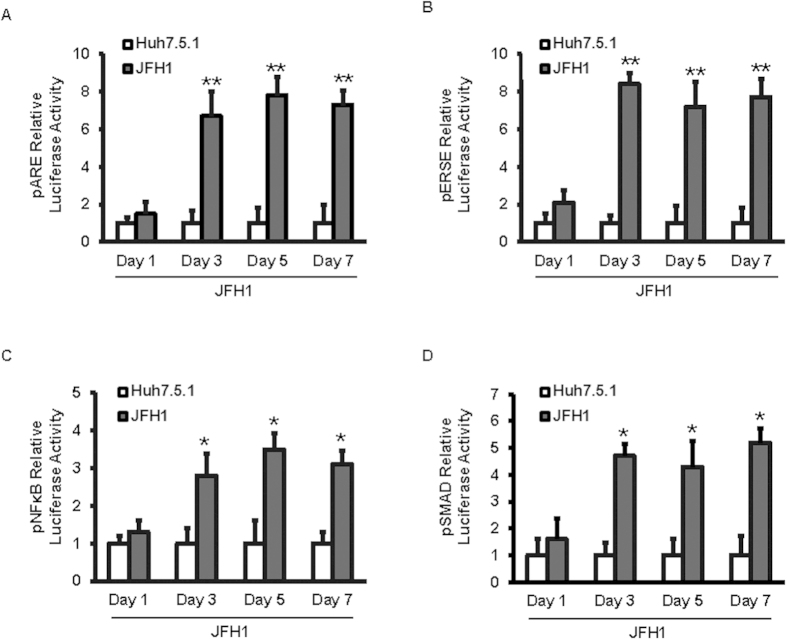
HCV infection induced antioxidant response, ER stress, NFκB, and TGF-β1 in a time dependent manner. Huh7.5.1 cells were infected with JFH1 HCV virus. After 24, 72, 120, and 168 hours of infection cells were transfected with plasmid pARE-luciferase, pERSE-luciferase, pNFκB-luc or pSMAD-luciferase reporter. The pRL-TK expressing *Renilla* luciferase was co-transfected as an internal control. ER stress pathway was monitored by a dual luciferase reporter assay system at 24 hours after transfection. HCV infection increased Nrf2/1, ERSE, NFκB, and SMAD luciferase signaling by 6.7, 8.4, 2.8, and 4.7-fold, respectively compared with un-infected Huh 7.5.1 cells (Fig. 1A–D). Relative luciferase activity (RLA) was normalized by dividing the firefly luciferase value by the *Renilla* luciferase value. *p < 0.05; and **p < 0.01 compared with un-infected Huh7.5.1 cells. Empty bar: Huh 7.5.1 cells, Black bar: JFH1-infected Huh 7.5.1 cells.

The induction of ROS, ER stress, NFκB, and TGF-β1 in JFH1 HCV-infected Huh7.5.1 cells was examined and compared to uninfected cells. Cells were infected with JFH1-HCV and viral replication was assessed by qRT-PCR. We studied whether HCV infection induced ROS, ER stress, NFκB, and TGF-β1 signaling. We measured the effects of JFH1-HCV on Nrf2/1, ERSE, NFκB, and TGF-β1-mediated transcriptional activity using a luciferase reporter construct containing the corresponding functional binding site. HCV infection increased pARE luciferase signaling in HCV-infected Huh7.5.1 cells by 6.7-fold compared with un-infected Huh7.5.1 cells at day 3 of infection (p = 0.007; Fig. 1A). The pARE reporter was designed to measure the transcriptional activity of the Nrf2 and Nrf1 transcription factors. Nrf2 and Nrf1 act as sensors for oxidative stress and direct transcription from the antioxidant response element (ARE). HCV increased pERSE-luciferase signaling in JFH1 cells by 8.4-fold compared with Huh7.5.1 cells at day 3 of infection (p = 0.003; Fig. 1B). The ERSE reporter monitored activity of the endoplasmic reticulum (ER) stress pathway. HCV infection increased NFκB signaling in JFH1 cells by 2.8-fold compared with Huh7.5.1 cells (p = 0.022; Fig. 1C). The NFκB-responsive luciferase construct encodes the firefly luciferase reporter gene under the control of a CMV promoter and tandem repeats of the NFκB transcriptional response element (TRE). HCV infection increased pSMAD luciferase signaling in HCV-infected Huh7.5.1 cells by 4.7–fold compared with un-infected Huh7.5.1 cells (p = 0.013; Fig. 1D). The SMAD reporter construct was designed to monitor the activity of TGF-β-induced signal transduction pathways. TGF-β signaling induces phosphorylation and activation of the SMAD2 and SMAD3 proteins, which then form complexes with SMAD4 and translocate to activate the expression of TGFβ-responsive genes.

### HCV infection activated each of the three arms of the UPR

**Figure 2:**
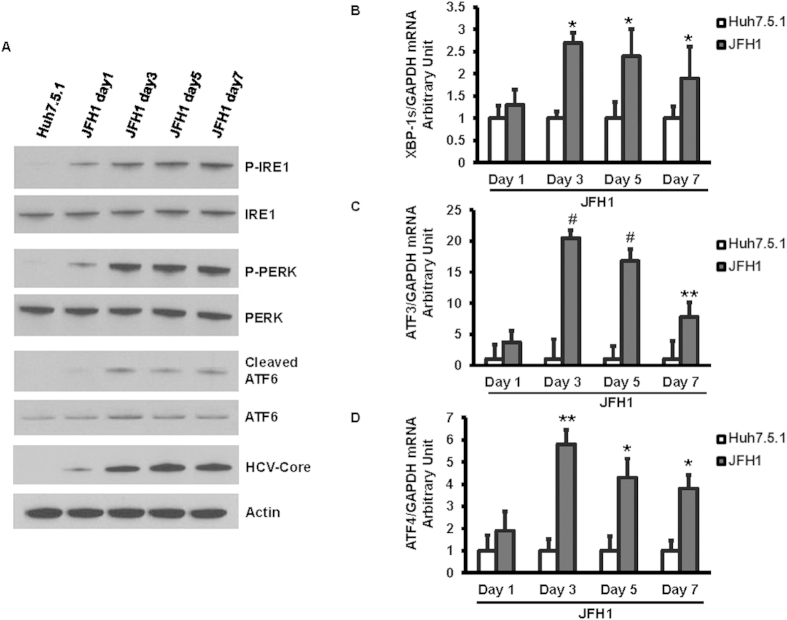
HCV infection activated each of the three arms of the UPR in a time dependent manner. Huh 7.5.1 cells were infected with HCV-JFH1. Following 24, 72, 129, and 168 hours of infection, protein was extracted at the indicated time points. The expression of phospho-IRE1, phospho-PERK, and cleaved ATF6 were analyzed by western blot. JFH1 HCV infection increased levels of phospho-IRE1, phospho-PERK, and the cleaved active form of ATF6 (Fig. 2A). Gene expression levels of spliced XBP-1, ATF3, and ATF4 at day 1, 3, 5, and 7 of infection were determined by real-time PCR and normalized to GAPDH. We found that the spliced XBP-1 mRNA level in JFH1-infected Huh7.5.1 cells increased by 2.7-fold at day 3 post infection compared to uninfected Huh7.5.1 cells (Fig. 2B). The spliced ATF3, and ATF4 mRNA levels were elevated by 20.5, 5.8-fold, respectively, in JFH1-infected Huh7.5.1 cells at day 3 post-infection compared to uninfected Huh7.5.1 cells (Fig. 2C,D). *p < 0.05; **p < 0.01; and #*p* < 0.001 compared between JFH1-infected and un-infected Huh7.5.1 cells. Empty bar: Huh 7.5.1 cells, Black bar: JFH1-infected Huh 7.5.1 cells.

We next examined the induction of the UPR in uninfected or JFH1 HCV-infected Huh7.5.1 cells over a 7-day time course. Each of the three arms of the UPR was activated in a wave-like pattern. We found that JFH1-HCV infection increased levels of the cleaved active form of ATF6 that peaked at day 3. The IRE1 pathway was also induced in JFH1-infected cells. Phosphorylated PERK was also detected in Huh7.5.1 cells infected with JFH1 (Fig. 2A). The level of spliced XBP-1s mRNA was significantly elevated at day 3 compared to uninfected cells (Fig. 2B). Similarly, mRNA levels of ATF3 and ATF4 were up-regulated by 20.5 and 5.8 fold, respectively, at the same time point compared to uninfected cells (Fig. 2C,D) (p = 0.001, 0.008). These data indicate that HCV induces each of the three arms of the UPR, including ATF6, IRE1α, and PERK.

### ROS and JNK inhibitors block HCV up-regulation of ER stress and UPR activation

**Figure 3:**
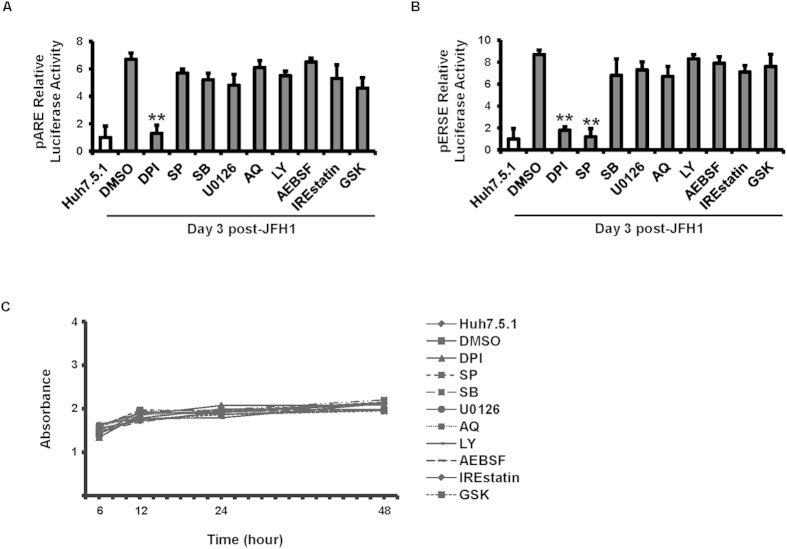
ROS and JNK inhibition decreased HCV-induced ER stress and UPR activation. Huh7.5.1 cells and Huh7.5.1 infected with JFH1 for 3 days were transfected with plasmid pARE or pERSE-luciferase reporter. The pRL-TK expression *Renilla* luciferase was co-transfected as an internal control. After 24 hours of transfection, cells were treated with several pathway inhibitor including DPI, SB, SP, U0126, LY, AEBSF, IREstatin, and GSK at 20 μM each. 1% DMSO was used as a negative control. ARE-mediated Nrf2 and ER stress signaling pathway were monitored by dual luciferase reporter assay at 24 hours after inhibitor treatment. Relative luciferase activity (RLA) was normalized by dividing the firefly luciferase value by the *Renilla* luciferase value. DPI (ROS inhibitor) decreased HCV-induced ARE signaling in JFH1-infected Huh7.5.1 cells. DPI and SP (JNK inhibitor) blocked HCV-induced ERSE signaling in JFH1-infected cells (Fig. 3A,B). There was no significant difference in proliferation between untreated and inhibitor treated cells at 6, 12, 24, and 48 hours of the treatment (Fig. 3C). **p < 0.01 for comparison of indicated treatment to the JFH1-infected cells in DMSO.

**Figure 4:**
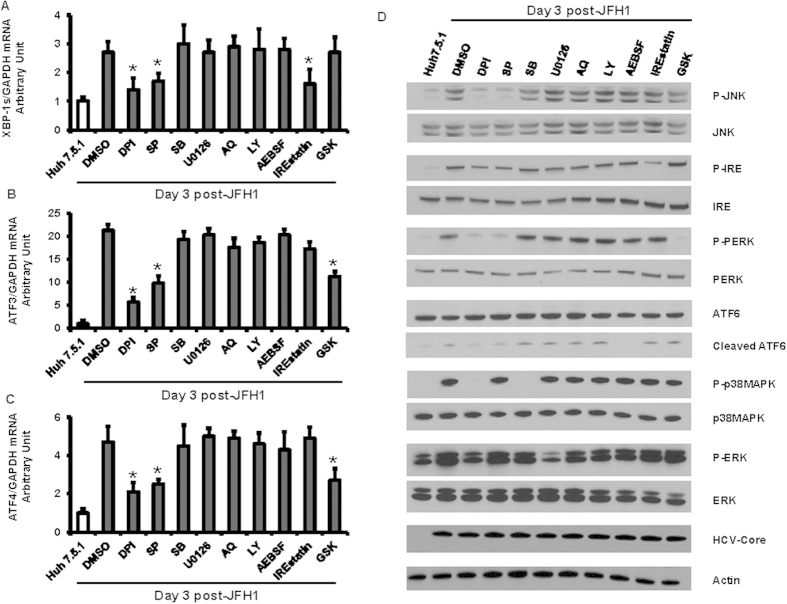
ROS and JNK inhibition decreased HCV-induced ER stress and UPR activation. Huh7.5.1 cells and Huh7.5.1 infected with JFH1 for 3 days were treated with several pathway inhibitor including DPI, SB, SP, U0126, LY, AEBSF, IREstatin, and GSK at the concentration 20 μM for 24 hours. 1% DMSO was used as a negative control. Gene expression levels of spliced XBP-1, ATF3, and ATF4 were determined by real-time PCR and normalized to GAPDH. We found that HCV-induced XBP-1s mRNA expression enhancement reduced by DPI, SP, and IREstatin treatments in JFH1-infected Huh7.5.1 cells compared with DMSO control. HCV-induced ATF3 and ATF4 mRNA expression enhancement was inhibited by DPI, SP, and GSK treatments, compared with DMSO control (Fig. 4A–C). *p < 0.05 for comparison of indicated treatment to the JFH1-infected cells in DMSO. Whole cell lysates were analyzed by western blot to measure phospho-JNK, phospho-IRE1, phospho-PERK, cleaved-ATF6, phospho-p38MAPK, and phospho-ERK. DPI decreased the HCV-induced phosphorylation of JNK, IRE1, PERK, p38MARK, ERK and the cleaved form of ATF6. SP blocked HCV-induced IRE1, PERK phosphorylation and ATF cleavage (Fig. 4D). We added 20 μg of protein to each well to assure equal protein loading in each lane. *Lane 1*, Huh7.5.1 + DMSO; *lane 2*, JFH1 + DMSO; *lane 3*, JFH1 + DPI; *lane 4*, JFH1 + SP; *lane 5*, JFH1 + SB; *lane 6*, JFH1 + U0126; *lane 7* JFH1 + AQ; *lane 8*, JFH1 + LY; *lane 9*, JFH1 + AEBSF; *lane 10*, JFH1 + IREstatin; *lane 11*, JFH1 + GSK.

To determine how HCV induces the ER stress and UPR pathways, we assessed the activity of ARE and ERSE reporters in un-infected or JFH1-infected cells treated with several different pathway inhibitors. DPI, a blocker of ROS formation, decreased HCV-induced ARE and ERSE signaling, compared with JFH1-infected Huh7.5.1 cells (Fig. 3A,B, p = 0.014, and 0.018, respectively). The JNK inhibitor SP also blocked ERSE signaling in JFH1-infected Huh7.5.1 cells (Fig. 3B, p = 0.005). None of the tested inhibitors had an effect on cell viability (Fig. 3C). Additionally, HCV-induced XBP1s, ATF3 and ATF4 mRNA expression were reduced by DPI and SP (Fig. 4A–C, respectively). At the protein level, the ROS inhibitor DPI decreased the HCV-induced phosphorylation of p38MAPK, JNK, ERK, IRE1, PERK and the cleaved form of ATF6. Furthermore, the JNK inhibitor SP blocked HCV-induced IRE1, PERK phosphorylation and ATF6 cleavage (Fig. 4D). These data indicate that HCV promotes ROS generation and subsequently activates ER stress through the JNK pathway. We also found that IREstatin reduced HCV-induced XBP-1 (p = 0.047), and GSK inhibited HCV-induced ATF3 and ATF4 mRNA (p = 0.044, and 0.045, respectively) enhancement. These findings suggest that IRE1 is in the upstream of XBP-1 and PERK is upstream of ATF3 and ATF4.

### ROS, JNK and IRE1 inhibitors block HCV-activated NFκB and TGF-β1

**Figure 5:**
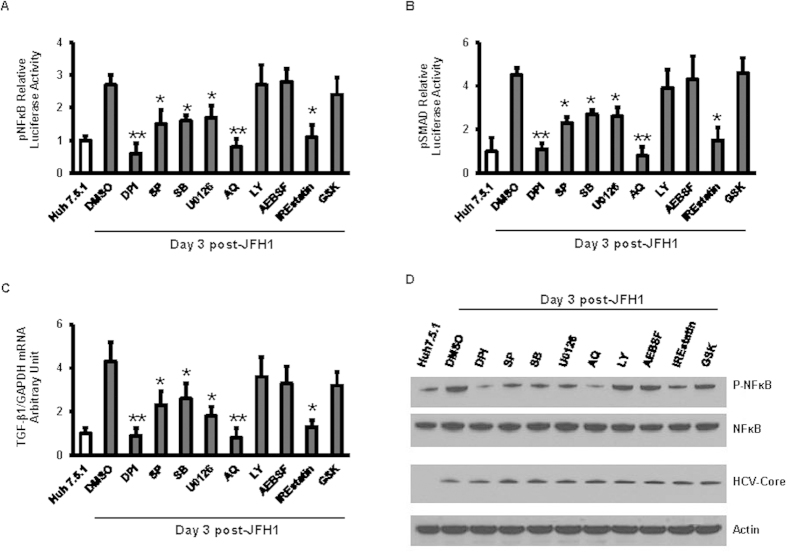
ROS, JNK, and IRE1 inhibitor decreased HCV activated NFκB and TGF-β1. Huh7.5.1 cells and Huh7.5.1 infected with JFH1 for 3 days were transfected with plasmid pNFκB or pSMAD-luciferase reporter for 24 hours. The pRL-TK expression *Renilla* luciferase was co-transfected as an internal control. After the transfection, cells were treated with several pathway inhibitor including DPI, SB, SP, U0126, LY, AQ, AEBSF, IREstatin and GSK (20 μM). 1% DMSO was used as a negative control. Luciferase signaling was monitored by a dual luciferase reporter assay at 24 hour after inhibitor treatment. Relative luciferase activity (RLA) was normalized by dividing the firefly luciferase value by the *Renilla* luciferase value. DPI, SB, SP, U0126, AQ, or IREstatin treatment decreased HCV-induced NFκB and TGF-β1 promoter signaling compared with DMSO control in JFH1 cells (Fig. 5A,B). TGF-β1 mRNA level was determined by real-time PCR and normalized to GAPDH. We found that HCV-induced TGF-β1 mRNA expression enhancement was blunted by DPI, SP, SB, U0126, AQ or IREstatin treatment compared with DMSO control in JFH1 cells (Fig. 5C). Whole cell lysates were analyzed by western blot to detect NFκB phosphorylation. DPI, SP, SB, U0126, AQ and IREstatin decreased HCV induced NFκB phosphorylation in JFH1 cells (Fig. 5D). *Lane 1*, Huh7.5.1 + DMSO; *lane 2*, JFH1 + DMSO; *lane 3*, JFH1 + DPI; *lane 4*, JFH1 + SP; *lane 5*, JFH1 + SB; *lane 6*, JFH1 + U0126; *lane 7* JFH1 + AQ; *lane 8*, JFH1 + LY; *lane 9*, JFH1 + AEBSF; *lane 10*, JFH1 + IREstatin; *lane 11*, JFH1 + GSK. *p < 0.05 and **p < 0.01 for comparison of indicated and JFH1-infected cells.

The effects of several different pathway inhibitors on NFκB and TGFβ-mediated transcriptional activity were measured using a luciferase reporter construct in JFH1-HCV-infected and uninfected Huh7.5.1 cells. We found that ROS inhibitor DPI and the NFκB inhibitor AQ each completely blocked HCV-induced NFκB and TGF-β1 induced-SMAD signaling (Fig. 5A, p = 0.007, and 0.035, respectively; Fig. 5B, p = 0.008, and 0.040, respectively). SP, SB, U0126 and IREstatin partially reduced HCV-induced NFκB signaling (p = 0.004, 0.047, 0.045 and 0.029, respectively) and partially reduced HCV-induced TGF-β signals (p = 0.043, 0.031, 0.033 and 0.025, respectively). Furthermore, HCV-induced TGF-β1 mRNA expression enhancement was completely blocked by DPI or AQ, and partially inhibited by SP, SB, U0126 and IREstatin (Fig. 5C, p = 0.006, 0.044, 0.020). Additionally, immunoblotting showed that DPI and AQ each completely blocked HCV-induced NFκB phosphorylation, SP, SB, U0126, and IREstatin partially decreased JFH1-induced NFκB phosphorylation (Fig. 5D). Taken together, our data suggest that IRE1 plays a partial role in the induction of NFκB and TGF-β1 during HCV infection. These data show that HCV activates NFκB and TGF-β1 through ROS production and partial through induction of JNK and the IRE1 pathway.

### siRNA-mediated knockdown of JNK and IRE1 inhibited JFH1 HCV activated ER stress, NFκB, and TGF-β1 luciferase activity

**Figure 6:**
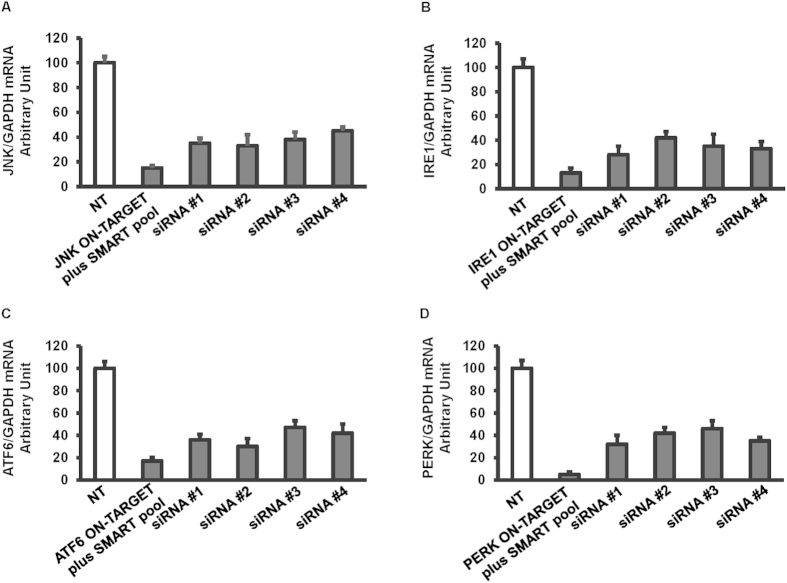
The comparison of ON-TARGET plus SMART pool siRNA and individual siRNA-mediated knockdown of JNK, IRE1, ATF6, and PERK. Huh7.5.1 cells were transfected with 25 nM small interference RNA (siRNA) against JNK, IRE1, ATF6 or PERK. After 48 hours of transfection, JNK, IRE1, ATF6, and PERK mRNA levels were determined by real-time PCR and normalized to GAPDH. The ON-TARGET plus SMART pool siRNAs had stronger mRNA knockdown effects for JNK, IRE1, ATF6, or PERK compared to its individual siRNA (Fig. 6A–D).

**Figure 7:**
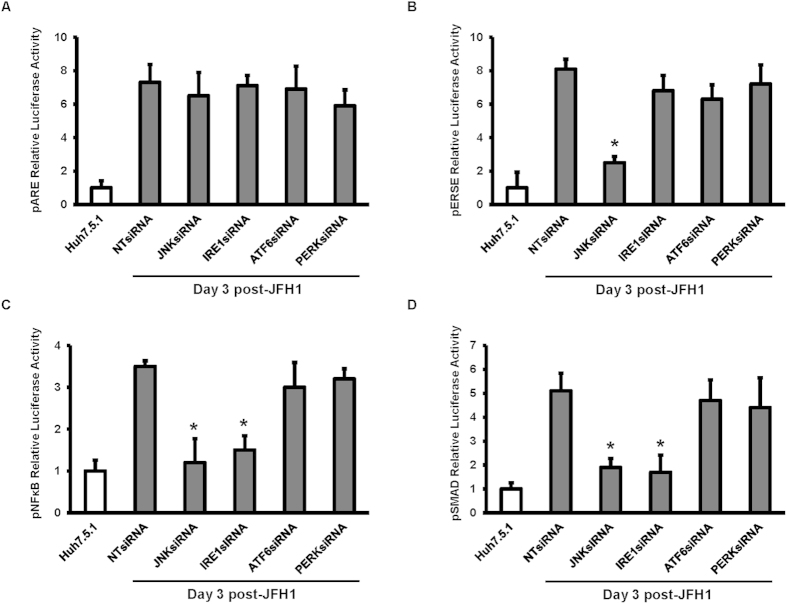
siRNA-mediated knockdown of JNK and IRE1 inhibited JFH1 HCV-activated ER stress, NFκB and TGF-β1 luciferase activity. Huh7.5.1 cells and Huh7.5.1 infected with JFH1 for 3 days were transfected with 25 nM small interfering RNA (siRNA) against JNK, IRE1, ATF6 or PERK. After 48 hours of transfection, cells were transfected with pARE, pERSE, pNFκB or pSMAD-luciferase reporter. ARE-mediated Nrf2, ERSE, pNFκB or pSMAD signaling were monitored by a dual luciferase reporter assay system at 24 hours after luciferase reporter transfection. We found that ARE-mediated Nrf2 signaling was not blocked by JNK, IRE1, ATF6 or PERK-specific knockdown (Fig. 7A). ERSE luciferase signaling was blocked by JNK siRNA (Fig. 7B). The siRNA-mediated knockdown JNK and IRE1 inhibited HCV-activated NFκB and SMAD luciferase activity in JFH1 cells compared to uninfected Huh7.5.1 cells (Fig. 7C,D). *p < 0.05 for comparison of indicated siRNA-mediated knockdown and JFH1-infected cells.

Based on our data above, we sought to further assess whether IRE1 was responsible for the induction of NFκB and TGF-β1 in HCV infection. To accomplish this, luciferase reporter experiments were performed to analyze levels of Nrf2/1, ERSE, NFκB, and TGF-β1-mediated transcriptional activity after siRNA-mediated knockdown of JNK, IRE1, ATF6, and PERK in Huh7.5.1 cells either mock-infected or infected with HCV. To confirm that HCV induces NFκB and TGF-β1 expression through a ROS production-JNK-IRE1 pathway, the ROS, ER stress, NFκB, and TGF-β1 promoter-driven luciferase activity were monitored in JFH1-infected cells transfected with siRNA-mediated knockdown of JNK, IRE1, ATF6, and PERK. We found that ON-TARGET plus SMART pool human siRNAs exhibited stronger mRNA knockdown effects compared to individual siRNA species (Fig. 6A–D). We found that ARE signaling was not affected by siRNA against JNK, IRE1, ATF6, and PERK (Fig. 7A). ERSE signaling was completely blocked by siRNA against JNK (Fig. 7B, p = 0.022). NFκB and TGF-β1 luciferase signaling were partially blocked by JNK and IRE1 specific siRNAs (Fig. 7C, p = 0.026, and 0.038, respectively; Fig. 7D, p = 0.032, and 0.025, respectively). In contrast, the ROS, NFκB, and TGF-β1 responsive reporters were not blocked by ATF6 and PERK-specific knockdown (Fig. 7A–D), indicating that ATF6 and PERK are independent of TGF-β1 induction.

### siRNA-mediated knockdown of JNK and IRE1 blocks HCV up-regulation of NFκB and TGF-β1 activation

**Figure 8:**
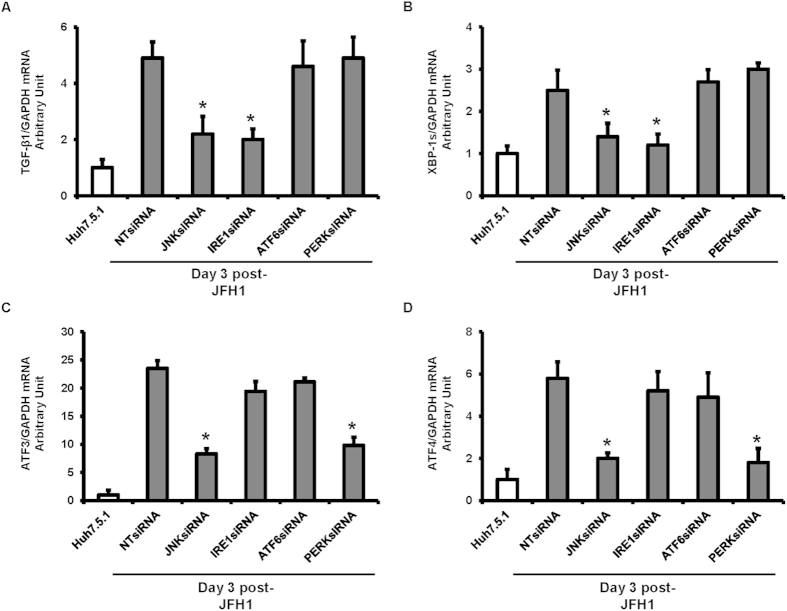
siRNA mediated knockdown of JNK, IRE1 blunted HCV up-regulation of NFκB and TGF-β1 activation. Huh7.5.1 cells and Huh7.5.1 cells infected with JFH1 for 3 days were transfected with 25 nM small interference RNA (siRNA) against JNK, IRE1, ATF6, and PERK for 48 hours. The mRNA expression levels of TGF-β1, spliced XBP-1, ATF3, and ATF4 were determined by real-time PCR and normalized to GAPDH. We found that JNK siRNA or IRE1 siRNA blunted HCV-induced TGF-β1 and XBP-1s mRNA enhancement in JFH1-infected Huh7.5.1 cells (Fig. 8A,B). JNK siRNA or PERK siRNA decreased HCV-induced ATF3 and ATF4 mRNA expression enhancement in JFH1-infected Huh7.5.1 cells (Fig. 8C,D). *p < 0.05 for comparison of indicated siRNA-mediated knockdown and JFH1-infected cells.

**Figure 9:**
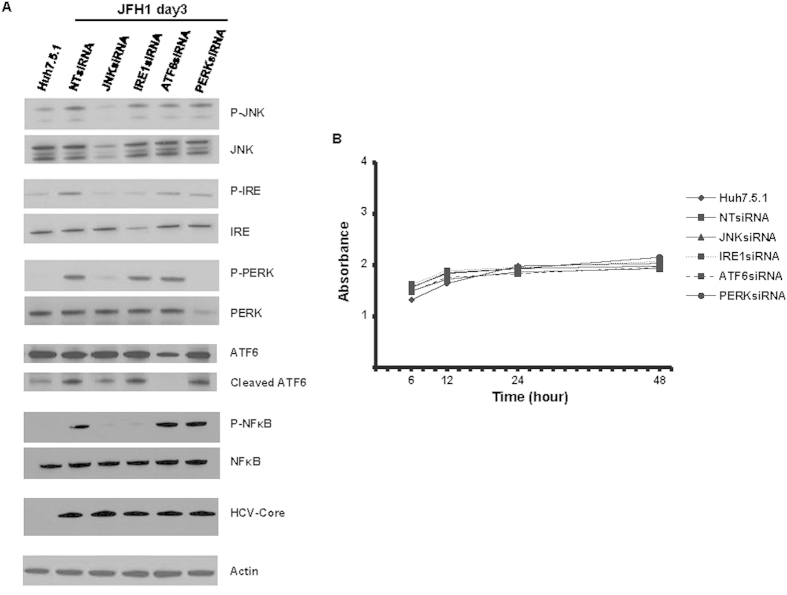
siRNA mediated knockdown of JNK, IRE1 blunted HCV up-regulation of NFκB activation. Huh7.5.1 cells and Huh7.5.1 cells infected with JFH1 for 3 days were transfected with 25 nM small interference RNA (siRNA) against JNK, IRE1, ATF6, and PERK. After 48 hours, the expression of phospho-JNK, phospho-IRE1, phospho-PERK, ATF6 cleavage and phospho-NFκB were analyzed by western blot. JNK siRNA decreased JFH1-induced IRE1, PERK, NFκB protein phosphorylation and ATF6 activation. IRE1 knockdown decreased HCV-induced NFκB phosphorylation in JFH1-infected Huh7.5.1 cells (Fig. 9A). We added 20 μg of protein to each well to assure equal protein loading in each lane. *Lane 1*, Huh7.5.1; *lane 2*, JFH1 + NTsiRNA; *lane 3*, JFH1 + JNKsiRNA; *lane 4*, JFH1 + IRE1siRNA; *lane 5*, JFH1 + ATF6siRNA; *lane 6*, JFH1 + PERKsiRNA. We found that siRNA knockdown of the tested genes did not affect cell viability compared to negative control siRNA (Fig. 9B).

RNA interference experiments were performed to assess the effects of JNK, IRE1, ATF6, and PERK knockdown on XBP1s, ATF3, ATF4, and TGF-β1 mRNA levels. As expected, JNK and IRE1 silencing partially decreased HCV-induced TGF-β1 (Fig. 8A, p = 0.039, and 0.035, respectively), and JNK siRNA blocked HCV-induced XBP1s, ATF3, and ATF4 mRNA expression enhancement in JFH1-infected cells (Fig. 8B–D). We found that IRE1 specific siRNA completely inhibited XBP1s mRNA expression but had no effect on ATF3 and ATF4 mRNA expressions. PERK specific siRNA did not affect TGF-β1 and XBP-1 mRNA levels (Fig. 8A,B), but reduced ATF3 and ATF4 mRNA expression, suggesting that PERK is upstream of ATF3 and ATF4. In contrast, ATF6 and PERK siRNAs did not affect TGF-β1, XBP1s, mRNA expression, indication that ATF6 and PERK are independent of the TGFB1 and ER stress pathways. We further examined the phosphorylation state of JNK1, IRE1, PERK, NFκB, and ATF6 activation in HCV-infected JNK, IRE1, ATF6, and PERK knockdown Huh7.5.1 cells. As expected, immunoblotting showed that JNK siRNA decreased JFH1-induced IRE1 phosphorylation, PERK phosphorylation, ATF6 activation, and NFκB phosphorylation. We found that each of the testes siRNAs did not affect cell viability (Fig. 9B). Additionally, IRE1 knockdown decreased NFκB phosphorylation. The siRNA-mediated knockdown of ATF6 and PERK did not affect NFκB activation (Fig. 9A). Taken together, these findings indicate that HCV induces NFκB and TGF-β1 through a ROS production-JNK-IRE1 dependent pathway.

## Discussion

HCV replication induces the formation of a membrane associated replication complex. Its replication activates ER stress and induces the UPR. HCV uses ER stress and manipulates the cellular response to ER stress to promote its persistence and pathogenesis. HCV-induced cellular responses may contribute to chronic liver disease by modulating cell proliferation, altering lipid metabolism, and potential oncogenic pathways. NFκB activation is triggered by oxidative stress and ER stress in cells that support HCV replication^24^. Our previous work has demonstrated that HCV induces ROS and TGF-β1 expression. HCV-mediated TGF-β1 enhancement occurs through a ROS-induced and p38 MAPK, JNK, ERK1/2, NFκB-dependent pathway^22^,^23^. TGF-β1 expression has been implicated in the pathogenesis of liver fibrosis^25^. However, whether and how HCV-induced ER stress, UPR activation, and TGF-β1 production are linked has not been established. The objective of our study was to determine whether HCV induces TGF-β1 production through ER stress and UPR activation pathways using the cell culture infectious JFH1 HCV model.

In this study, we discovered that HCV induces ER stress and activates all three arms of the UPR through c-Jun N-terminal kinase (JNK). Additionally, we found that during HCV infection, the IRE1-XBP1 pathway is activated, as demonstrated by the presence of spliced XBP1 mRNA. We found that knockdown of JNK and IRE1-JFH1 cells decreased the expression of NFκB and TGFβ1. Given that the activation of the IRE1-XBP1 pathway correlates with expression of NFκB and TGF-β1 in JFH1-HCV-infected cells, it therefore appears plausible that signaling through this pathway is important during HCV infection and implicates its involvement in HCV pathogenesis. To this end, it has been shown that UPR induced by HCV positively regulates HCV RNA replication in a cell culture system supporting efficient HCV propagation as abrogation of one of the three known UPR pathways suppresses HCV replication^26^. It is possible that the UPR induced by HCV perpetuates viral infection by relieving cells from stress conditions and facilitating replication. Additionally, we found that ER stress partially contributes to HCV-induced NF-κB activation and enhancement of TGF-β1 expression via the IRE1 arm of the UPR. HCV infection has been linked to ER stress, which is associated with ROS production in patients with chronic infection^27^. Reactive oxygen species can induce the proliferation of hepatic stellate cells (HSCs) and release of TGF-β1. Multiple studies have reported that ROS mediates activation of the p38 MAPK, JNK, ERK, and NFκB pathways^22^. Our study provides new evidence, using an infectious tissue culture model, that HCV induces ER stress and the UPR in a JNK-dependent manner. ER stress and UPR activation partially contribute to HCV-induced NFκB activation and enhancement of TGF-β1 expression.

**Figure 10:**
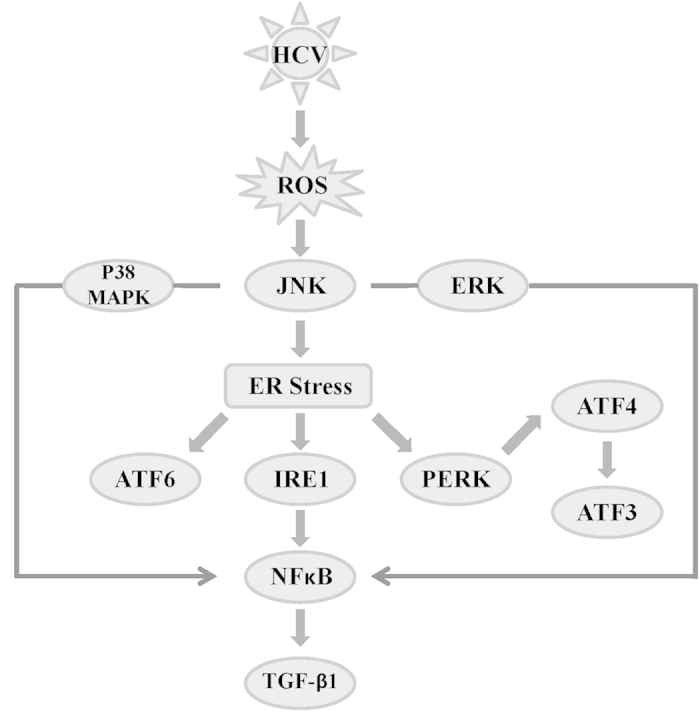
Proposed model by which HCV infection induces NFĸB and TGF-β1 through ER stress and the UPR pathway in a JNK-dependent manner. HCV induces ROS induction, which in turn activates the phosphorylation of JNK. The phosphorylated JNK subsequently induces ER stress. The activation of one arm of UPR pathway, IRE1, induces the phosphorylation of NFκB. The activated NFκB is translocated to nucleus and up-regulates TGF-β1 expression.

We therefore propose a model in which HCV induces ROS induction, which in turn activates the phosphorylation of JNK. Phosphorylated JNK subsequently induces ER stress. The activation of one arm of the UPR pathway, IRE1, in turn induces the phosphorylation of NFκB. The activated NFκB translocates to nucleus and up-regulates TGF-β1 expression (Fig. 10). This study provides insight into the intracellular signaling events in HCV infected cells and extends our knowledge regarding the mechanisms of ER stress, the UPR-pathway and TGF-β1 activation. Our results further suggest that strategies to counteract HCV-associated liver pathogenesis could be directed against key intracellular events (ER stress and oxidative stress) induced by HCV.

## Methods

### Cell Culture

The human hepatocellular carcinoma cell line, Huh 7.5.1, was obtained from Dr. Frank Chisari, Scripps Institute, CA. Huh 7.5.1 cells were grown in Dulbecco’s modified Eagle’s medium (DMEM) supplemented with 10% fetal calf serum and 100 units of penicillin/ml.

### HCV infection

Cell supernatant containing the infectious JFH1 HCV virus was used to infect naïve Huh 7.5.1 cells at appropriate dilutions (moi = 0.2) for luciferase reporter assays and RNA interference studies as previous descriptions^28^,^29^,^30^.

### Reagents/Inhibitors

To study the impact of HCV infection on ER stress, NFκB, and TGF-β1 induction, inhibitors of intermediate steps were tested, including inhibitors of ROS (diphenyliodonium; DPI), p38 MAP kinase (SB203580; SB), JNK (SP600125; SP), ERK1/2 (U0126), PI3K (LY294002; LY), NFκB activation (6-amino-4-(4-phenoxyphenylethylamino; AQ), ATF6 (AEBSF), PERK (GSK2606414; GSK). These compounds were obtained from Calbiochem, San Diego, CA. The IRE1 inhibitor Irestatin 9389 was obtained from AxonMedchem, Netherlands. The inhibitors stock solution was dissolved in 1% dimethyl sulfoxide (DMSO) and 1% used as a negative control. The cells were incubated with appropriate inhibitors at a final concentration of 20 μM for 24 hours.

### Luciferase Reporter Assays

Uninfected and HCV-infected Huh 7.5.1 cells were transfected with Cignal Antioxidant Response, ERSE, and SMAD Response Luciferase Reporters (SABiosciences, Frederick, MD) by using Fugene HD transfection reagent (Roche, Indianapolis, IN) according to the manufacturer’s protocol. NFκB signaling was quantified using an NFκB promoter construct expressing firefly luciferase (pNFκB-Luc) and a construct expressing *Renilla* luciferase (pRL-TK) as an internal background control. The ARE reporter was designed to measure the transcriptional activity of the Nrf2 and Nrf1 transcription factors. Nrf2 and Nrf1 act as sensors for oxidative stress and direct transcription from the antioxidant response element (ARE). The ERSE reporter construct monitored activity of the endoplasmic reticulum (ER) stress pathway. The ERSE is a motif that mediates the transcriptional response to ER stress. The NFκB reporter construct was designed to monitor the activity of NFκB-regulated signal transcription pathways. The NFκB-responsive luciferase construct encodes the firefly luciferase reporter gene under the control of a CMV promoter and tandem repeats of the NFκB transcriptional response element (TRE). The SMAD reporter construct was designed to monitor the activity of TGF-β-induced signal transduction pathways. TGF-β signaling induces phosphorylation and activation of the SMAD2 and SMAD3 proteins, which then form complexes with SMAD4 and translocate to activate the expression of TGFβ-responsive genes. Cells were harvested and cellular lysates were analyzed for luciferase reporter assay kit (Promega, Madison, WI). Relative luciferase activity (RLA) was normalized by dividing the firefly luciferase value by the *Renilla* luciferase value.

### RNA interference

Uninfected infected and JFH1 HCV-infected Huh7.5.1 cells were transfected with small interfering RNA (siRNA) against JNK, IRE1, ATF6, and PERK. A non-targeting siRNA was used as a negative control. The Dharmacon ON-TARGET plus SMART pool Human siRNAs (25 nM final concentration) (Fisher Scientific Life Science Research, Pittsburgh, PA) used for gene knock-down. The transfection protocol was performed according to the manufacturer’s instructions. Seventy-two hours after transfection knock-down efficiency was assessed by real-time PCR and Western blotting from lysate of siRNA-transfected cells.

### Analysis of Gene Expression by Quantitative Real-time PCR

Total cellular RNA was isolated using the QIA Shredder and RNeasy kit (Qiagen, Valencia, CA), as described by the manufacturer’s protocol. Reverse transcription was performed using Applied Biosystems high-capacity cDNA reverse transcription kits (Invitrogen, Carlsbad, CA) with random primers. To quantify gene expression, quantitative real-time PCR was performed in the Bio-Rad IQ5 system (Bio-Rad Laboratories, Hercules, CA) using Finnzymes SYBR green I dye (New England Biolabs, Ipswich, MA), and sequence-specific primers, as indicated in Table 1. The glyceraldehyde-3-phosphate dehydrogenase (GAPDH) gene was used as an internal control. The reactions were performed under the following conditions: 95 °C for 3 min, followed by 45 cycles at 94 °C for 20 seconds, 60 °C for 30 seconds, and 72 °C for 20 seconds. The mRNA level of each gene was normalized to GAPDH levels to obtain mRNA arbitrary units (fold change).

### Western Blot Analysis

Cells were washed with phosphate-buffered saline (PBS) and lysed in radioimmunoprecipitation assay (RIPA) buffer (50 mM Tris-HCl [pH7.4], 150 mM NaCl, 1% NP-40, 0.5% Sodium deoxycholate, 0.1% SDS, 5 mM EDTA) containing protease and phosphatase inhibitor cocktail (Sigma-Aldrich, St. Louis, MO) as previous description^31^,^32^. Cell lysates were sonicated and cleared by centrifugation at 14,000 rpm for 15 min at 4 °C. Protein concentration was determined and samples were boiled at 95 °C for 5 min in SDS-PAGE sample buffer. Twenty micrograms of protein were loaded into each well and were separated on a NuPAGE Novex precast 4-to-12% gradient Bis-Tris gels (Invitrogen, Carlsbad, CA) using the SDS-PAGE technique. The proteins were transferred to nitrocellulose membranes and blocked with 5% bovine serum albumin (BSA) in TBS/Tween. The blotted membranes were probed overnight at 4 °C with specific primary antibodies. The secondary antibodies used were horseradish peroxidase (HRP)-conjugated ECL donkey anti-rabbit IgG and HRP-conjugated ECL sheep anti-mouse IgG (Amersham Biosciences, Piscataway, NJ). Chemiluminescence detection of the bands was performed using the ECL Western blotting detection kit (Amersham Biosciences, Piscataway, NJ). Membranes were probed with anti-actin (Sigma-Aldrich, St. Louis, MO) to confirm equal loading.

### Cell proliferation assay

Cell proliferation was analyzed by using the Cell Counting Assay Kit-8 (CCK-8) (Dojindo Molecular Technologies, Gaithersburg, MD) according to the manufacturer’s protocol. At 6, 12, 24, or 48 hours post inhibitor treatment or transfection, the cells were washed with PBS. Ten microliters of CCK-8 solution was added to each well, the cells were incubated for 1 hour, and the absorbance at 490 nm was measured using the automatic multiwall spectrophotometer (Bio-Rad, Richmond, CA). Experiments were performed in triplicate with representative data presented.

### Antibodies

Each of the primary antibodies were used according to the manufacturer’s protocol: Phospho-JNK #4671, JNK #9252, IRE1α #3294, PERK #5683, phospho-p38 MAPK #9215, p38 MAPK #9212, phospho-p44/42 MAPK #9106, p44/42 MAPK #4695, (Cell Signaling, Danvers, MA). phospho- IRE1α #PA1-16927, phospho-PERK #MA5-15033, and HCV core (Thermo Scientific, Rockford, IL). ATF6 #ab11909 (Abcam, Cambridge, MA).

### Statistical Analysis

Statistical analysis was conducted using the 2-tailed Student *t* test. Data are expressed as means ± standard deviations (S.D.). Triplicate determinations were performed in all real-time experiments, and all experiments were repeated at least three times.

## Additional Information

**How to cite this article**: Chusri, P. *et al.* HCV induces transforming growth factor β1 through activation of endoplasmic reticulum stress and the unfolded protein response. *Sci. Rep.* **6**, 22487; doi: 10.1038/srep22487 (2016).

## Figures and Tables

**Table 1:** List of primers used for real time PCR.

Gene Name	Forward Primer 5′- 3′	Reverse Primer 5′- 3′
**XBP1s**	CTGAGTCCGCAGCAGGTG	TGCCCAACAGGATATCAGACT
**ATF3**	TTGCAGAGCTAAGCAGTCGTG	ATGGTTCTCTGCTGCTGGGATTCT
**ATF4**	CCTTCGACCAGTCGGGTTTG	CTGTCCCGGAAAAGGCAT
**TGF-β1**	GGCCAGATCCTGTCCAAGC	GTGGGTTTCCACCATTAGCAC
**JFH1**	TCTGCGGAACCGGTGAGTA	TCAGGCAGTACCACAAGGC
**GAPDH**	ACAGTCCATGCCATCACTGCC	GCCTGCTTCACCACCTTCTTG
